# Affinity of Tau antibodies for solubilized pathological Tau species but not their immunogen or insoluble Tau aggregates predicts in vivo and ex vivo efficacy

**DOI:** 10.1186/s13024-016-0126-z

**Published:** 2016-08-30

**Authors:** Erin E. Congdon, Yan Lin, Hameetha B. Rajamohamedsait, Dov B. Shamir, Senthilkumar Krishnaswamy, Wajitha J. Rajamohamedsait, Suhail Rasool, Veronica Gonzalez, Josien Levenga, Jiaping Gu, Charles Hoeffer, Einar M. Sigurdsson

**Affiliations:** 1 Departments of Neuroscience and Physiology, New York University School of Medicine, Medical Science Building, MSB459, 550 First Avenue, New York, NY 10016 USA; 2 Department of Integrative Physiology, Institute for Behavioral Genetics, University of Colorado, Boulder, CO 80309 USA; 3 Departments of Psychiatry, New York University School of Medicine, New York, NY 10016 USA

**Keywords:** Alzheimer’s disease, Tau protein, Paired helical filaments, Antibodies, Immunotherapy

## Abstract

**Background:**

A few tau immunotherapies are now in clinical trials with several more likely to be initiated in the near future. A priori, it can be anticipated that an antibody which broadly recognizes various pathological tau aggregates with high affinity would have the ideal therapeutic properties. Tau antibodies 4E6 and 6B2, raised against the same epitope region but of varying specificity and affinity, were tested for acutely improving cognition and reducing tau pathology in transgenic tauopathy mice and neuronal cultures.

**Results:**

Surprisingly, we here show that one antibody, 4E6, which has low affinity for most forms of tau acutely improved cognition and reduced soluble phospho-tau, whereas another antibody, 6B2, which has high affinity for various tau species was ineffective. Concurrently, we confirmed and clarified these efficacy differences in an ex vivo model of tauopathy. Alzheimer’s paired helical filaments (PHF) were toxic to the neurons and increased tau levels in remaining neurons. Both toxicity and tau seeding were prevented by 4E6 but not by 6B2. Furthermore, 4E6 reduced PHF spreading between neurons. Interestingly, 4E6’s efficacy relates to its high affinity binding to solubilized PHF, whereas the ineffective 6B2 binds mainly to aggregated PHF. Blocking 4E6's uptake into neurons prevented its protective effects if the antibody was administered after PHF had been internalized. When 4E6 and PHF were administered at the same time, the antibody was protective extracellularly.

**Conclusions:**

Overall, these findings indicate that high antibody affinity for solubilized PHF predicts efficacy, and that acute antibody-mediated improvement in cognition relates to clearance of soluble phospho-tau. Importantly, both intra- and extracellular clearance pathways are in play. Together, these results have major implications for understanding the pathogenesis of tauopathies and for development of immunotherapies.

**Electronic supplementary material:**

The online version of this article (doi:10.1186/s13024-016-0126-z) contains supplementary material, which is available to authorized users.

## Background

Tau immunotherapy was a logical approach following the success of amyloid-β (Aβ) immunotherapies in mouse models but faced resistance as tau was not thought to be accessible to antibodies. However, target engagement was feasible both intra- and extracellularly. Antibodies against tau and other targets have been detected intraneuronally [1, 2], and studies over the last several decades suggested that all amyloid diseases may be transmissible between cells under proper conditions [3]. Following our initial report of active tau immunotherapy leading to clearance of tau aggregates in transgenic mice with associated functional improvements, several studies by us and others have confirmed and extended these findings (reviewed in [4, 5]). Concurrently, spreading of tau pathology between cells in culture and via anatomically connected brain regions in animals has now been shown by several groups (reviewed in [6, 7]). A few phase I trials have now been initiated on active and passive tau immunotherapies [5]. The hope is that this approach may be more effective than targeting Aβ in the later stages of the disease as tau pathology correlates better with dementia than Aβ plaques [8].

Although the efficacy of tau immunotherapy has been confirmed in various models, our knowledge of the mechanisms involved is rather limited. Tau antibodies have now been detected intraneuronally in several studies by a few groups [1, 9–14] and such uptake shown to be necessary for acute tau clearance [10]. However, some antibodies do not appear to be taken up in appreciable amounts and are likely to primarily work extracellularly [15–17]. Such differences in uptake are well known in other immunotherapy fields and may be related to antibody charge [18, 19]. Several tau epitopes have been successfully targeted using a similar study design (reviewed in [5]). However, very limited knowledge exists regarding the ideal affinity of antibodies and which tau species they should bind to be effective in promoting clearance of pathological tau protein. It is conceivable that very high affinity antibodies, at least against certain epitopes, may promote tau assembly or prevent their disassembly.

Most recently, we have developed a novel set of monoclonal antibodies targeting the phospho-serine 396,404 region. Two of these, 4E6 and 6B2, enter neurons and co-localize with tau [11]. In brain slice cultures, both antibodies reduce soluble phospho-tau after 6 weeks of treatment, and 4E6 has been shown to acutely reduce tau levels in primary neurons via an intracellular mechanism [10, 11]. The two antibodies display different binding characteristics with 4E6 being phospho-selective and 6B2 having conformational properties influenced by phosphorylation and an apparent higher affinity for tau [11].

We tested the efficacy of these antibodies acutely in vivo and their ability to prevent toxicity, seeding and transmission of tau pathology in primary neuronal cultures, using paired helical filaments (PHF) isolated from an Alzheimer’s brain. In addition, we examined whether neuronal uptake of antibody was necessary for efficacy, and what role timing of antibody addition had on the observed mechanism of action. Our data indicates that 4E6 acutely improves spatial learning and memory, which is associated with a reduction in soluble phospho-tau protein. Furthermore, 4E6 prevents toxicity, seeding and transmission of tau pathology even though it binds poorly to most forms of tau, whereas 6B2 is ineffective although it binds strongly to most forms of tau. These unexpected results are likely to have major implications for the clinical development of tau immunotherapies, and can be explained by 4E6’s high affinity for solubilized PHF, whereas the ineffective 6B2 binds primarily to aggregated but not to solubilized PHF. Hence, affinity for particular forms of tau predict efficacy. Further, whether the antibody is working outside or inside the neuron depends on the timing of PHF and antibody addition. Antibodies with access to both intra- and extracellular pools of pathological tau protein are likely to be more efficacious than antibodies acting only within one compartment.

## Results

### In vivo studies

#### 4E6 acutely improves cognition in htau mice and reduces soluble phospho-tau protein, whereas 6B2 does not affect cognition or tau levels

At 11–12 months of age, the htau mice were assigned to control and treatment groups with similar cognitive (CFSM test) and motor status (Rotorod and Open Field). After baseline behavioral assessment, the mice received three antibody injections over a two week period and were re-evaluated on the same tests in addition to a fear conditioning test, followed by brain extraction for tissue analysis.

#### Behavior

**Fig. 1:**
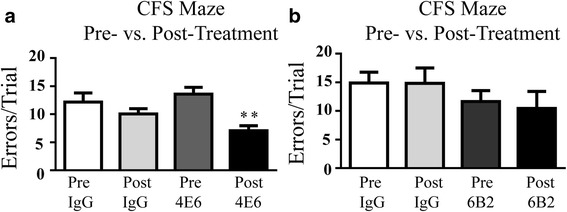
Acute 4E6- but not 6B2 therapy improved spatial learning and memory in htau mice. **a** 4E6 immunized mice showed significant improvements in Closed Field Symmetrical (CFS) Maze (48 % fewer errors, *p* < 0.01), compared to their pre-immunization performance, whereas control IgG treated mice did not improve. When divided by gender, both males and females treated with 4E6 showed significant improvement over their pre-treatment performance (52 and 44 % fewer errors, *p* < 0.01 and *p* < 0.05, respectively, see text for average and SEM) Repeated measures, two-way ANOVA revealed a significant effect of treatment (*p* = 0.0018) but not of gender (*p* = 0.5145), indicating that the results seen are not attributable to gender differences. In contrast, animals treated with IgG did not show improvement when all animals were considered together, or when divided by gender. **b** 6B2 treated mice also did not improve in the same test. **: *p* < 0.01

**Fig. 2:**
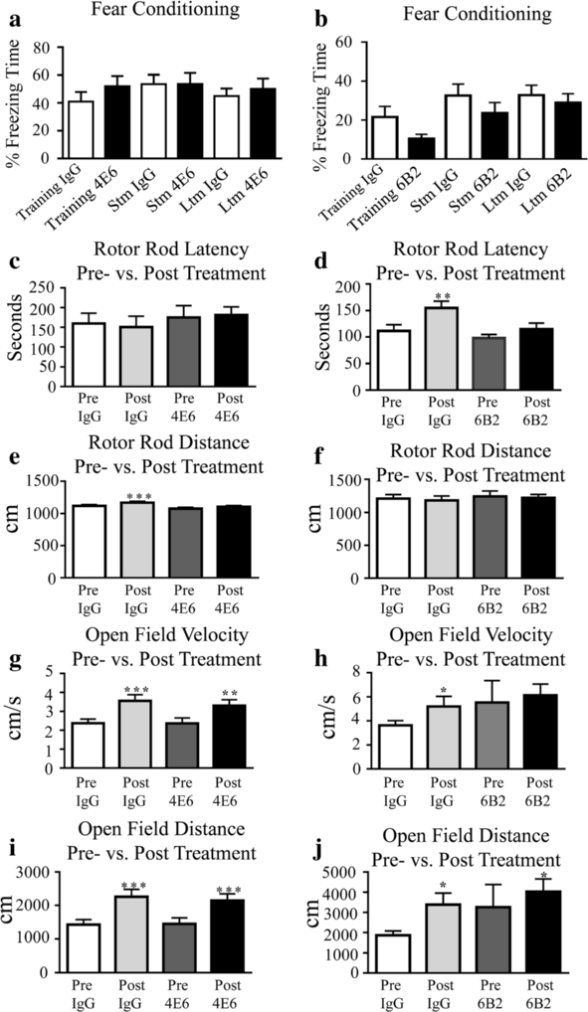
Neither tau antibody led to benefits in a fear conditioning test or affected motor performance. **a**–**j** Treatment benefits were not observed in a fear conditioning test for **a** 4E6 or **b** 6B2, which relies on different brain circuits than the navigational test, or in motor tests (**c**-**j**), which were performed to verify that the cognitive benefits cannot be explained by confounding changes in motor performance. As expected, mice from both treated and control groups generally performed better on their post-treatment motor tests because of their pre-treatment training. *: *p* < 0.05, **: *p* < 0.01, ***: *p* < 0.001. Stm: Short-term memory, Ltm: Long-term memory

As shown by trial errors, acute treatment with 4E6 led to significant improvements in spatial learning and memory in the CFSM test (48 % fewer errors in post-test vs. pre-test; 13.6 (average errors) ± 1.2 (SEM) to 7.1 ± 0.9, *p* < 0.01), whereas the IgG control mice did not improve compared to their pre-injection performance (Fig. 1a) These differences were not gender related. 4E6 treated males (52 % fewer errors; 14.6 ± 0.8 to 7.0 ± 1.4, *p* < 0.01) performed similar to 4E6 treated females (44 % fewer errors; 12.7 ± 2.2 to 7.1 ± 1.2, *p* < 0.05). Repeated measures, two-way ANOVA revealed a treatment effect (*p* = 0.0018), but not a gender effect (*p* = 0.5145). Conversely, 6B2 treatment did not result in improvements in this test (Fig. 1b). Neither antibody showed benefits in a fear conditioning test (Fig. 2a, b). Both treatment groups of mice performed similarly to IgG controls in motor function tests (rotor rod and open field tests, Fig. 2c–j), suggesting that the 4E6-mediated improvements in cognitive functions were direct results of the immunization, but not secondary effects from motor function changes.

#### Immunohistochemistry, western and immunoblot analyses

**Fig. 3:**
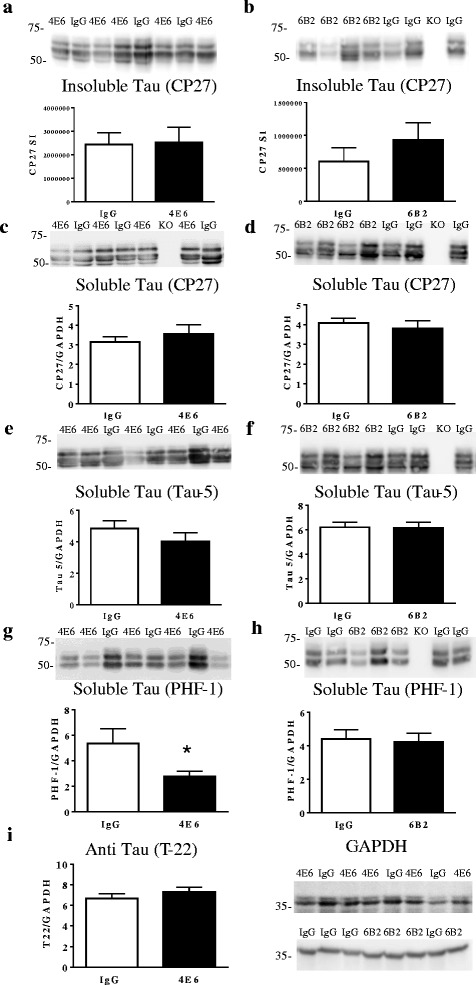
4E6, but not 6B2, reduced soluble phospho-tau levels in htau mice. **a**, **b** Insoluble tau protein (sarkosyl pellet) levels were not altered in **a** 4E6 or **b** 6B2 immunized mice as detected by total human tau antibody CP27, compared to IgG controls. Similar results were obtained with total tau antibody Tau-5 or phospho-tau antibody PHF-1 (not shown, see values in text). **c–f** Likewise, soluble tau levels (low speed supernatant; CP27, Tau-5) normalized to GAPDH were not significantly altered in 4E6 treated mice compared to IgG control group. **g, h** Animals treated with 4E6 showed a significant reduction in levels of soluble PHF-1 reactive tau relative to IgG controls (48 % reduction, *p* = 0.037), while those treated with 6B2 showed no change. This beneficial effect of the therapy was not gender related (two-way ANOVA; gender effect: *p* = 0.905). **i** Also, the cognitive benefits in the 4E6 group could not be explained by differences in T22 detected oligomeric tau as those levels did not differ between the 4E6 and IgG group (dot blot quantitation shown, similar results were seen on Western blots (not shown). *: *p* < 0.05

Rather mild tau pathology was detected in PHF-1 or MC1 stained brain sections (Additional file 1: Figure S1). PHF-1 stained sections had some neuropil staining but lacked cell body staining. Likewise, MC1 staining was limited, although a few intraneuronal tau aggregates were evident at high magnification. This is as expected as MC1 detects earlier tau pathology than PHF-1. There were no apparent differences between the treatment groups. Overall, we have observed a slower development, and less extensive pathology in this model compared to the initial report [20]. The observed differences in pathology may be due to several factors, such as a reduction in the transgene copy number, promoter methylation, or the cleanliness of the facility the animals are housed in. There may also be selection effects, where animals with less pathology produce larger litters and become overrepresented in the colony. Other researchers have observed a lessening of pathological severity over time, or spontaneous loss of phenotype in transgenic lines [21, 22]. Likewise, no significant differences were seen via Western blot using CP27 in either the total tau low speed supernantant, or in sarkosyl insoluble tau for either antibody (Fig. 3a–d). Tau-5, an additional total tau antibody, also showed no significant differences (Fig. 3e, f). However, acute 4E6 treatment significantly reduced soluble PHF-1 reactive tau (48 % reduction; *p* = 0.037, Fig. 3g), whereas 6B2 did not (Fig. 3h) . This beneficial effect of the therapy was not gender related (two-way ANOVA; gender effect (*p* = 0.905), and did not appear to be oligomer specific as T22 did not reveal any differences between the 4E6 and IgG groups (Fig. 3i). Sarkosyl insoluble fractions were also probed with PHF-1 and Tau-5, with no significant differences seen between antibody and IgG treated animals for either 4E6 or 6B2. Average chemiluminescent signal for PHF-1 in animals treated with 4E6 and control IgG were 582,276 ± 138,812 and 491,638 ± 165,340 (*p* = 0.50), and for Tau-5 616,980 ± 151,750 and 509,180 ± 154,157 (*p* = 0.67), respectively. The same analyses were performed for 6B2 and IgG treated mice with average chemiluminescent signal for PHF-1 being 327,492 ± 92,674 and 322,075 ± 84,285 (*p* = 0.84) and for Tau-5 567,725 ± 179,647 and 549,867 ± 159,106 (*p* = 0.86), respectively.

For all blots prepared using low speed supernatant samples, chemiluminescent signal was normalized using GAPDH (Fig. 3). There was minor variation in some samples, but GAPDH blots showed no significant difference between the control and treated groups. Two bands are visible on the representative blot prepared from the 4E6 study, whereas only one band is visible in the 6B2 study. This is likely due to differences in running time between blots.

#### Mechanistic in vitro and ex vivo studies

To further clarify the mechanisms of the positive effects of 4E6 and lack thereof for 6B2, various in vitro and ex vivo experiments were performed.

#### Incubation with AD-derived PHF induces toxicity in primary neurons and 4E6, but not 6B2, prevents these effects

**Fig. 4:**
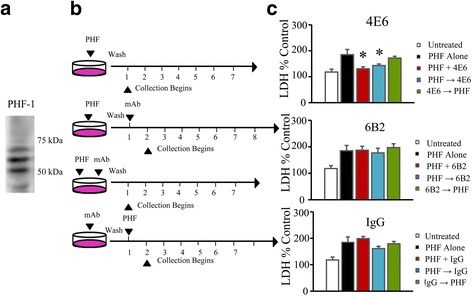
PHF Characterization, dosing methods, and PHF induced toxicity as measured via LDH. **a** Immunoblot showing enriched human PHF tau (PHF-1 staining) derived from an Alzheimer’s brain. **b** Neurons were exposed to PHF under one of four dosing paradigms. PHF was added alone, 24 h prior to antibody addition (PHF → Ab), together with antibody (PHF + Ab), or 24 h after antibody (Ab → PHF). Cells were washed with Neurobasal media between each step, and collection began 24 h after the last treatment applied. **c** In cells treated with 10 μg/ml PHF, LDH signal averaged 67 % above that of untreated controls (*p* < 0.01). 4E6 in the PHF + Ab and PHF → Ab paradigms significantly reduced LDH compared to PHF alone, and were comparable to untreated samples (11 and 15 % above control, *p* < 0.05), indicating that the antibody prevented toxicity. However, the Ab → PHF was not effective in reducing LDH signal (53 % above control) and showed no significant improvement over PHF alone samples. **d** All samples treated with 6B2 showed significantly higher levels of LDH relative to untreated controls (69, 59 and 79 % above control for the PHF + Ab, PHF → Ab, and Ab → PHF treatment groups respectively, *p* < 0.05). None of the treatments with 6B2 reduced LDH relative to PHF alone. **e** IgG was also not effective in preventing the increased LDH levels triggered by the addition of PHF. LDH in the PHF + Ab, PHF → Ab, and Ab → PHF groups was increased to 80, 43 and 61 % above control values (*p* < 0.05). None of the groups were significantly different from PHF alone. *: *p* < 0.05, **: *p* < 0.01, ***: *p* < 0.001

Primary neuronal cultures were prepared from JNPL3 mice and exposed to human derived PHF in one of four dosing conditions (Fig. 4a, b): PHF alone, PHF added 24 h before antibody (PHF → Ab), PHF material and antibody added concurrently (PHF + Ab), or antibody was added 24 h before PHF (Ab → PHF). PHF was used at 1 and 10 μg/ml. Its solubility was verified at the 10 μg/ml. No visible pellet was seen at this concentration after 100,000 x g centrifugation for 60 min. Prior work by others indicates that PHF can be soluble at least up to 100 μg/ml [23].

#### Lactate dehydrogenase assay

Untreated control cells showed an 18 % increase in LDH over 7 days, indicating that some normal cell loss occurs.

##### PHF 10 μg

Results from one-way ANOVA showed significant treatment effect (*p* = 0.002; Fig. 4d). Addition of 10 μg/ml PHF resulted in a significant increase in LDH levels relative to control samples at day 7 (a 67 % increase above control, *p* < 0.01). In contrast, when 1 μg/ml of 4E6 was added to the cultures together with the PHF material (PHF + Ab group) or 24 h after PHF (PHF → Ab), this increase was reduced from 67 % above control to 12 and 15 %, respectively (*p* < 0.05 relative to PHF alone). In both treatment paradigms, the groups were not significantly different from control cells. However, LDH levels in the Ab → PHF group were significantly higher than in the untreated cells (53 % above control, *p* < 0.05), and did not differ significantly from PHF alone treated samples. Hence, the Ab → PHF treatment approach for 4E6 was ineffective, in contrast to the PHF → Ab and PHF + Ab paradigms. In addition, all of the 6B2 treatment groups had significantly higher LDH levels relative to untreated controls (69, 59 and 79 % above control for the PHF + Ab, PHF → Ab, and Ab → PHF treatment groups respectively, *p* < 0.05). None of the 6B2 groups differed significantly from PHF alone samples, indicating that the 6B2 antibody was ineffective under any of the paradigms in preventing PHF toxicity. IgG1 control did not influence PHF toxicity under any of the treatment conditions, again confirming the specificity of the 4E6 effect to prevent PHF toxicity.

##### PHF 1 μg

As for 10 μg PHF, a one-way ANOVA revealed significant treatment effect (*p* = 0.03; data not shown). At the 1 μg/ml dose of PHF, LDH levels were again, as with the higher dose, significantly higher than in the untreated control cells although the toxicity was not as severe (35 % above control, *p* < 0.05). As in the higher dose samples, 4E6 in either the PHF + Ab or PHF → Ab paradigms significantly reduced LDH levels relative to PHF alone samples (7 and 15 % below control values, *p* < 0.05 for both). IgG1 control did not significantly influence PHF toxicity under any of the treatment conditions (data not shown).

#### NeuN immunoblotting

**Fig. 5:**
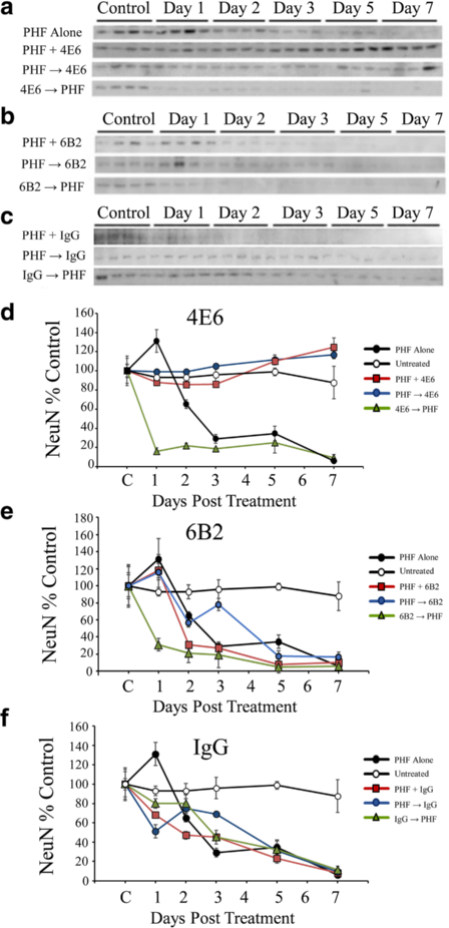
4E6, but not 6B2, prevented neurotoxicity induced by 10 μg/ml PHF. **a**–**c** NeuN immunoblots from samples treated with 10 μg/ml PHF alone and with PHF and 1 μg/ml **a** 4E6, **B** 6B2 or **c** control mouse IgG1. **d** Quantitation of signal in samples treated with PHF alone and a combination of PHF and 4E6. At 10 μg/ml, PHF alone reduced NeuN levels by 94 % relative to untreated control cells (*p* < 0.0001). PHF + Ab and PHF → Ab paradigms resulted in NeuN levels significantly higher than those incubated with PHF alone (116 and 124 % control, *p* < 0.0001 for both) and were comparable to control levels. However, the Ab → PHF paradigm was ineffective and showed a significantly reduced NeuN signal relative to control (93 % loss, *p* < 0.0001) and no improvement over PHF alone samples. **e** 6B2 did not prevent NeuN loss under any of the dosing conditions used. None of the groups were significantly improved relative to PHF alone. **f** Similar to the 6B2 samples, the control IgG1 did not protect against the PHF induced loss of NeuN and these samples were similar to the PHF alone group

In addition to LDH signal, toxicity was also examined via immunoblotting with an antibody recognizing neuronal marker NeuN (Fig. 5a-c). For these and all subsequent immunoblots, untreated control cells served as internal control because typical markers could not be used due to the PHF toxicity.

##### PHF 10 μg

Results from ANOVA showed a significant effect of both treatment and time (*p* < 0.0001 for both). When incubated with 10 μg/ml PHF, NeuN signal steadily declined and was reduced 94 % relative to untreated control samples by day 7 (Fig. 5d). As was the case with the LDH samples, the PHF + Ab and PHF → Ab paradigms for 4E6 (1 μg/ml) prevented PHF toxicity (samples were 16 and 24 % above untreated control on day 7, Fig. 5d) and did not significantly differ from untreated control at any time point. Also as above, the Ab → PHF dosing method was not effective in preventing the loss of NeuN over the treatment period (93 % loss), at each time point showed NeuN levels significantly lower than control (*p* < 0.0001) and showed no improvement over the PHF alone samples. As in the LDH assay, neither 6B2 (Fig. 5e) nor IgG1 (Fig. 5f) had any effect on PHF induced toxicity, and did not significantly differ from PHF alone.

##### PHF 1 μg

**Fig. 6:**
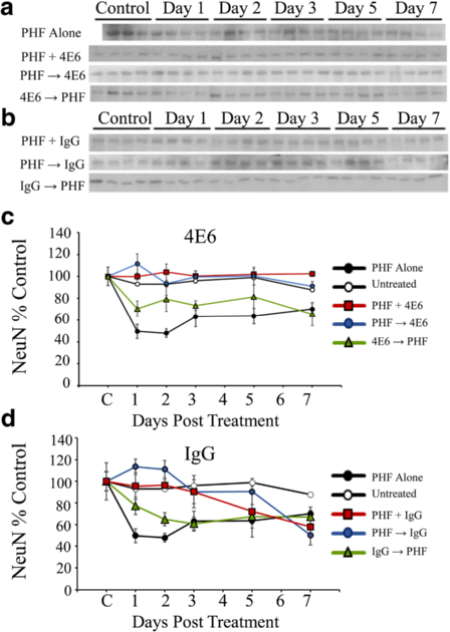
Low dose PHF (1 μg/ml) reduced NeuN signal, and 4E6 prevented this toxicity. **a**, **b** NeuN immunoblots for samples treated with 1 μg/ml PHF alone or 1 μg/ml PHF and 1 μg/ml **a** 4E6 or **b** control IgG1. **c** Chemiluminescent signal was quantified and as in the higher dose samples, exposure to PHF decreased NeuN signal (30 % loss relative to untreated cells, *p* < 0.001) after 7 days. For 4E6, the Ab → PHF group also showed a decline in NeuN levels (36 % decrease relative to control, *p* < 0.05) comparable to PHF alone samples. Again the PHF + Ab and PHF → Ab groups had significantly higher NeuN levels at the end of the experiment (102 and 91 % control values, *p* < 0.01 relative to PHF alone). **d** Mouse IgG1 was ineffective in preventing NeuN loss under all of the dosing conditions and did not differ significantly from PHF alone

Additional groups of neurons were incubated with 1 μg/ml PHF and 1 μg/ml of 4E6 or control IgG1 (Fig. 6) and also probed with NeuN (Fig. 6a, b). As with the higher PHF dose, the two way ANOVA results showed a significant effect of treatment and time (*p* < 0.0001 and 0.007). A 30 % loss of NeuN signal relative to untreated control cells was observed in the PHF alone group after seven days in culture. PHF alone samples were significantly lower than untreated controls at each time point (*p* < 0.01-0.001). As in the 10 μg PHF experiments, the PHF + Ab and PHF → Ab groups showed efficacy in preventing the loss of NeuN, and at day 7 were comparable to untreated controls and significantly higher that PHF alone samples (*p* < 0.01 for both at day seven, Fig. 6c). The Ab → PHF group also showed a decline in NeuN levels (36 % decrease over seven days relative to untreated controls, *p* < 0.05) comparable to PHF alone samples, confirming the inefficacy of this approach. As in the higher PHF dose samples, control mouse IgG1 (Fig. 6d) was ineffective in preventing toxicity under any of the dosing conditions.

Together, the LDH and NeuN data consistently show that tau antibodies can prevent the dose-dependent toxicity triggered by exposure to misfolded tau aggregates, but not all antibodies are effective. 4E6, but not 6B2, prevented toxicity, and then only under certain conditions. When 4E6 is added prior to PHF, it is possible that the relatively low level of tau native to the neurons does not provide sufficient targets to promote the retention of antibody that is necessary to protect the cells.

In addition to both of the quantitative measures used to assess toxicity, a qualitative visual inspection of the cells was made prior to each collection. In untreated cells, cell bodies appeared healthy with an extensive network of processes. In contrast, PHF treated cells had shrunken cell bodies, retracted processes, and debris from dead cells was clearly visible under the microscope. Further, media color provides an indication of the level of cellular respiration occurring. When cultures were healthy, the changing pH resulted in a shift towards a more orange color. However, in cultures where cells were dying, the media remained pink indicating limited cellular activity. These PHF-induced changes were prevented in 4E6-treated cultures.

### 4E6 treatment prevents changes in tau levels caused by PHF exposure, whereas 6B2 has no effect

#### Total Tau

##### PHF 10 μg

**Fig. 7:**
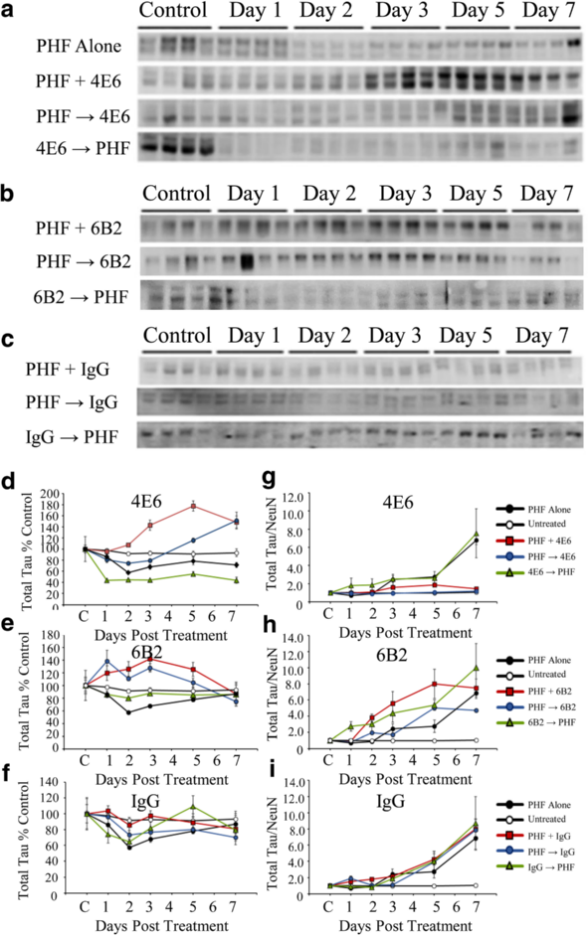
4E6, but not 6B2, prevented increase in the tau/NeuN ratio caused by exposure to 10 μg/ml PHF. **a–c** Immunoblots probed with a pan-tau antibody of samples incubated with PHF or PHF in combination with **a** 4E6, **b** 6B2 or **c** control IgG1. **d** Quantitation of total tau levels in samples incubated with PHF and 4E6. With PHF alone, total tau levels decreased relative to control before recovering (29 % decrease at day 7). In contrast, samples in the PHF + Ab and PHF → Ab groups had significantly higher tau levels (48 and 51 % above control, *p* < 0.001 relative to PHF alone). Ab → PHF samples did not significantly differ from PHF alone. **e** After 7 days in culture, samples treated with PHF and 6B2 were not significantly different than the PHF alone samples. **f** The IgG1 treated cells also did not differ significantly from PHF alone. **g** We then used the values obtained from the NeuN data to normalize tau levels. Due to the substantial toxicity seen using LDH and NeuN immunoblotting, tau levels alone did not provide an accurate picture of the effects of PHF exposure. Incorporating NeuN data allowed us to account for neuronal loss when assessing changes induced by PHF. Using this method, it became evident that the remaining cells in the PHF group had significantly more tau (tau/NeuN) that control cells (5.6 fold increase, *p* < 0.0001). In the PHF + Ab and PHF → Ab groups, adjusted tau levels were comparable to control and significantly lower than the PHF alone samples (*p* < 0.0001). **h** When NeuN levels were controlled for, all 6B2 treated groups had tau levels significantly higher that controls with no significant difference from PHF alone group (*p* < 0.05-0.0001). **i** Controlling for NeuN did not alter the pattern of results seen in cells incubated with IgG

In addition to measures of toxicity, the effect of PHF and antibody treatments on tau levels was also investigated via immunoblot (Fig. 7a–c). Similar to the NeuN results, a significant effect of both dosing method and time were seen (*p* < 0.0001 for both). At the 10 μg/ml concentration, the total tau levels in the PHF alone group showed an initial decrease followed by a recovery (Fig. 7). However, by day 7 total tau levels were reduced relative to control cells (a 29 % decrease, *p* < 0.05). In contrast, the 4E6 PHF + Ab and PHF → Ab groups had significantly higher tau levels (48 and 51 % above control, *p* < 0.01 relative to control, *p* < 0.001 relative to PHF alone, Fig. 7d) than PHF alone. Again the 4E6 Ab → PHF group, or any dosing paradigm of the 6B2 (Fig. 7e) and IgG1 (Fig. 7f) groups were ineffective in preventing changes in tau levels at day 7 compared to PHF alone cells.

##### Tau/NeuN

When these results were normalized using the NeuN values to take into account PHF toxicity, there were again significant effects of treatment and time by two-way ANOVA (*p* < 0.0001 for both). It became clear that the remaining cells in the PHF and 4E6 Ab → PHF groups had significantly more tau than control cells at day seven (a 5.6 and 5.5 fold increase at seven days, *p* < 0.0001 for both, Fig. 7g). In the 4E6 PHF + Ab and PHF → Ab groups, adjusted tau levels were comparable to untreated controls and significantly lower than the PHF alone samples (*p* < 0.0001 for both). All of the 6B2 (Fig. 7h) and IgG1 (Fig. 7i) dosing groups did not differ significantly from PHF alone, and were significantly higher than untreated cells by day 7 (*p* < 0.05-0.0001).

##### PHF 1 μg

**Fig. 8:**
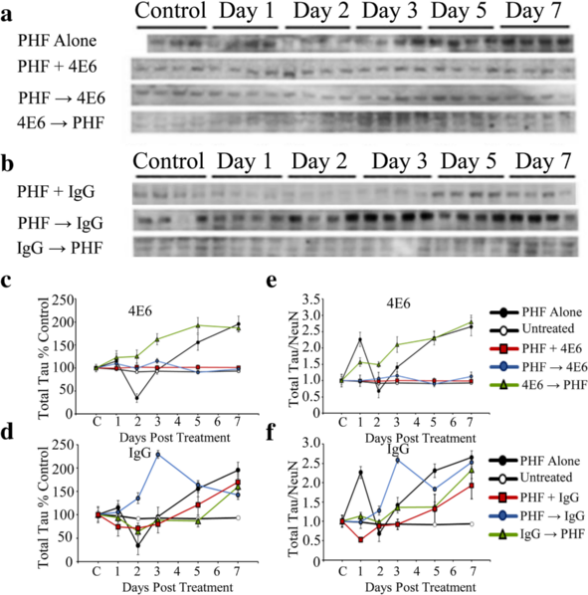
Low dose PHF (1 μg/ml) increased the intracellular tau/NeuN ratio, which was prevented by 4E6. **a**, **b** Immunoblots for samples exposed to PHF alone or with **a** 4E6 or **b** control IgG1, probed with a pan tau polyclonal antibody. **c** Quantitation of total tau levels shows that at 1 μg/ml, PHF promoted significant increases in intracellular tau (95 % above control, *p* < 0.0001). For 4E6, the PHF + Ab and PHF → Ab were significantly lower than the PHF alone samples (*p* < 0.0001). Ab → PHF cells were not reduced relative to PHF alone. **d** IgG did not prevent against significant increases in total tau after exposure to PHF. **e** As above, the ratio of tau/NeuN was determined, and again this ratio was significantly increased in PHF alone samples relative to control cells (1.6 fold increase, *p* < 0.0001). Neurons treated with 4E6 in the PHF + Ab and PHF → Ab, but not Ab → PHF groups had significantly lower corrected tau levels compared to the PHF alone groups (*p* < 0.0001). **f** When adjusted for NeuN levels tau levels in the PHF + Ab, PHF → Ab, and Ab → PHF control IgG groups were 0.98, 1.4 and 1.3 fold higher than control, and not significantly different from PHF alone

At the 1 μg/ml dose, the increased tau levels were evident even before normalization with NeuN level (Fig. 8a, b). Again a two-way ANOVA showed significant dosing and time effects (*p* < 0.0001 for both, Fig. 8c). PHF promoted significant increases in intracellular tau (95 % above control, *p* < 0.0001) by experimental day 7. The PHF + Ab and PHF → Ab were not changed relative to untreated control and were significantly lower than the PHF alone samples (*p* < 0.0001 for both). IgG1 (Fig. 8d) was ineffective under all of the dosing paradigms, as was the 4E6 Ab → PHF treatment group.

##### Tau/NeuN

Significant dosing and time effects were found using two-way ANOVA (*p* < 0.0001 for both). After normalizing tau levels with NeuN, again the PHF alone cells had significantly increased tau levels relative to control cells (1.6 fold increase, *p* < 0.0001 for both, Fig. 8e). Only the 4E6 PHF + Ab and PHF → Ab groups had significantly lower normalized tau levels compared to the PHF alone group and did not differ from untreated control samples (*p* < 0.0001 for both). After 7 days in culture, IgG1 samples did not significantly differ from PHF alone (Fig. 8f).

#### Phospho-Tau

##### PHF 10 μg

**Fig. 9:**
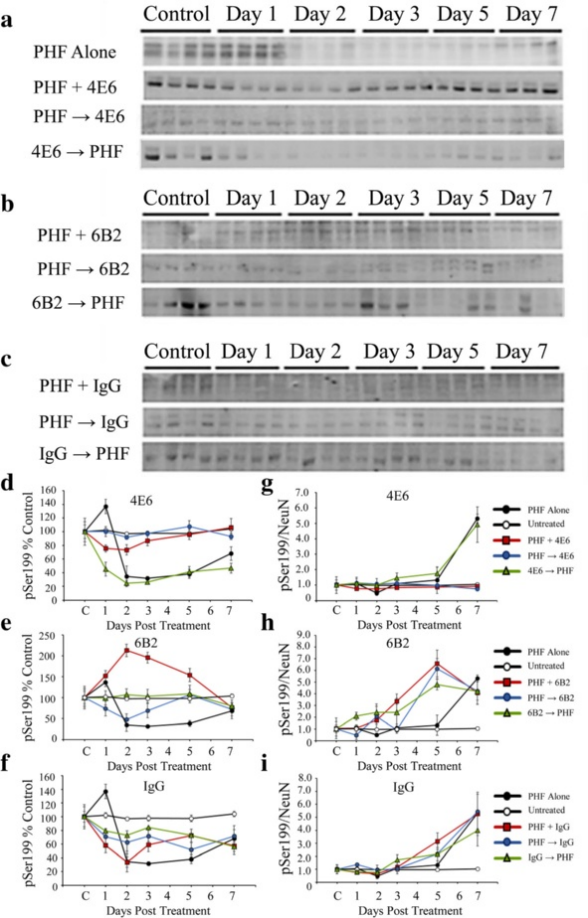
4E6, but not 6B2, prevented the increase in the phosphorylated tau/NeuN ratio caused by exposure to 10 μg/ml PHF. **a–c** Samples probed with a polyclonal antibody recognizing tau phosphorylated at Ser199 from cells exposed to PHF alone or PHF in combination with **a** 4E6, **b** 6B2 or **c** control IgG1. **d** PHF alone samples had significantly reduced phospho-tau levels relative to untreated cells (34 % reduction, *p* < 0.05). For 4E6, both the PHF + Ab and PHF → Ab treatment groups had significantly higher phospho-tau levels than the PHF alone group (*p* < 0.001 and 0.05 respectively). **e** In samples treated with a combination of PHF and 6B2, none of the treatment groups were significantly different from PHF alone. **f** As with 6B2, uncorrected IgG1 samples showed no significant difference relative to PHF alone. **g** Correcting for NeuN levels to take PHF toxicity into account, PHF alone samples had higher ratio of P-Ser199/NeuN, compared to untreated controls (a 4.1 fold increase, *p* < 0.0001). Phospho- tau levels in the 4E6 PHF + Ab and PHF → Ab groups were significantly lower than the PHF alone samples (91 and 78 % of untreated controls, *p* < 0.0001). **h** When corrected for NeuN levels, 6B2 treated cells were not significantly different from PHF alone samples. **i** When NeuN levels were considered, IgG1 samples were also not significantly different from PHF alone

In addition to total tau levels, we also assessed the levels of tau phosphorylated at Ser199 (Fig. 9a–c). Statistical analysis with two-way ANOVA revealed significant effect of dosing method and time (*p* < 0.0001 for both). Under the 10 μg/ml PHF conditions, PHF alone and 4E6 Ab → PHF samples had significantly reduced phospho-tau levels relative to untreated cells at day seven (34 and 54 % reduction, *p* < 0.05 and 0.001 respectively, Fig. 9d). However, both the PHF + Ab and PHF → Ab treatment groups had significantly higher phospho-tau levels that the PHF alone group (*p* < 0.001 and 0.05 respectively, Fig. 9d), and did not differ from untreated cells at day 7. None of the 6B2 (Fig. 9e) or IgG1 (Fig. 9f) dosing groups differed from PHF alone by experimental day 7.

##### Phospho-Tau/NeuN

Correcting for NeuN levels to take into account PHF toxicity, significant effects of dosing method and time were seen using two-way ANOVA (*p* < 0.0001 for both). PHF alone samples showed higher levels of P-Ser199 tau (4.1 fold increase at day 7, *p* < 0.0001). Phospho-tau levels in the PHF + Ab and PHF → Ab groups were significantly lower than the PHF alone samples and comparable to untreated controls (*p* < 0.0001 for both, Fig. 9g). As with the uncorrected values, none of the 6B2 (Fig. 9h) or IgG1 (Fig. 9i) groups, or the 4E6 Ab → PHF group, were different from PHF alone.

##### PHF 1 μg

**Fig. 10:**
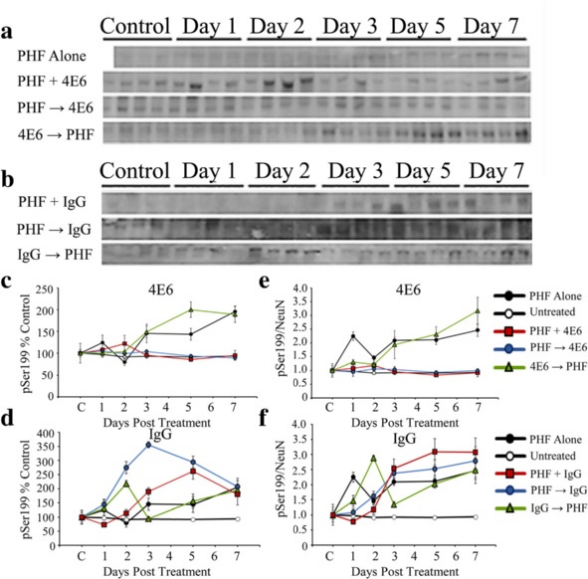
Low dose PHF (1 μg/ml) increased the phosphorylated tau/NeuN ratio, which was prevented by 4E6. **a**, **b** Immunoblots of samples treated with 1 μg/ml PHF alone or with **a** 4E6 or **b** IgG1, reacted with a P-Ser199 antibody. **c** In cells treated with 1 μg/ml PHF and 4E6, the PHF alone and Ab → PHF groups showed significantly higher phosphorylated tau levels compared to untreated control samples (165 and 185 % above control, *p* < 0.0001 for both). The PHF + Ab and PHF → Ab groups were significantly lower (91 and 112 % control, *p* < 0.0001 for both) than the PHF alone samples. **d** All of the IgG groups showed significantly higher average phospho-tau levels than the untreated controls (79, 119, and 94 % above control, *p* < 0.0001 for all) after 7 days. None of the groups were significantly different compared to the PHF alone samples. **e** As above, NeuN levels were used to control for cell loss, and the pattern of results seen in 4E6 treated cells remained. The PHF alone and Ab → PHF groups had significantly higher phospho-tau/NeuN ratios than untreated samples (1.46 and 1.9 fold higher, *p* < 0.0001). The PHF + Ab and PHF → Ab groups had significantly lower phospho-tau levels than the PHF alone samples comparable to untreated controls. **f** Correcting for NeuN levels, the same pattern of results in cells treated with control IgG was observed. All groups showed significantly higher levels of phospho-tau/NeuN relative to untreated controls (2, 3.9 and 1.5 fold increase in the PHF + Ab, PHF → Ab, and Ab → PHF groups, *p* < 0.0001 for all) and no difference relative to the PHF alone samples

ANOVA results revealed significant effects of dosing method and time (*p* < 0.0001 for both; see Fig. 10a, b for immunoblots). PHF alone samples under the 1 μg/ml dosing conditions had significantly higher phospho-tau levels than untreated control cells (65 % above control at day 7, *p* < 0.0001). Again, 4E6 was effective in preventing PHF-induced pathological changes under the PHF + Ab and PHF → Ab dosing conditions. Both groups had significantly lower phospho-tau levels than PHF alone at levels comparable to untreated controls (*p* < 0.0001 for both, Fig. 10c). However, under the Ab → PHF dosing conditions, 4E6 was ineffective at reducing phospho-tau levels. None of the IgG1 groups (Fig. 10d) differed compared to the PHF alone samples.

##### Phospho-Tau/NeuN

Controlled for NeuN levels, these differences remained. As above, dosing method and time produced significant effects by two-way ANOVA (*p* < 0.0001 for both). The phospho-tau levels in the PHF alone samples were 1.5 fold higher than that of the untreated control cells by experimental day seven (*p* < 0.0001). Of the 4E6 dosing paradigms, the PHF + Ab and PHF → Ab groups had tau levels comparable to untreated controls and significantly lower than those seen in the PHF alone samples (*p* < 0.0001 for both, Fig. 10e). As above, none of the IgG1 groups (Fig. 10f) differed from PHF alone samples.

Together, the total and phospho-tau data show that PHF addition to the cultured neurons increases the levels of these forms of tau in the neurons that survive PHF toxicity. The 4E6 antibody, in addition to preventing PHF-induced toxicity, prevents PHF-induced increases in both total- and phospho-tau, under the PHF + Ab and PHF → Ab conditions. As in the LDH and NeuN toxicity studies, 4E6 Ab → PHF and all three dosing conditions of 6B2 and IgG1 were ineffective.

#### Pattern of PHF and Antibody binding differs depending on dosing method

**Fig. 11:**
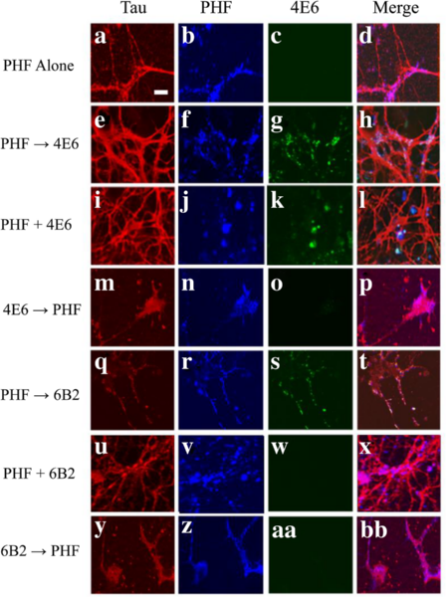
PHF and antibody binding differs depending on antibody and dosing method. Primary neurons were treated with 10 μg/ml human derived PHF material alone, PHF and 1 μg/ml 4E6 or 6B2 together (co-incubation), or 24 h PHF incubation followed by 4E6 or 6B2 for an additional 24 h. All coverslips were stained with Dako pan tau polyclonal antibody. **a**–**d** Neurons readily took up the PHF tau from the media and PHF positive puncta were detected in cell bodies and neuronal processes. **e**–**h** As shown above, PHF was taken up by the cultured neurons. 4E6 was added 24 h later and co-localized intracellularly with the exogenous PHF. **i**–**l** When added together, 4E6 and the PHF material formed large extracellular aggregates. **m**–**p** In the 4E6 → PHF group, PHF positive puncta were detected in the cells, but 4E6 was not. **q**–**t** When 6B2 was added 24 h after PHF, we observed intracellular co-localization. **u**–**x** Unlike 4E6, coincubation of 6B2 and PHF did not produce the large extracellular complexes. **Y**-**BB** When 6B2 was added before PHF, PHF but not 6B2 was seen in the cells

To further clarify these efficacy differences, fluorescently labeled PHF and 4E6 or 6B2 were utilized to examine whether the treatment paradigm affects the pattern and location of PHF and antibody binding. Primary neurons were incubated using the same dosing methods described above. Confocal images were collected 24 h after the last treatment was applied. PHF was readily taken up into neurons and could be seen throughout the cells after 24 h in culture (Fig. 11a–d). In cells from the PHF → Ab group, we also observed PHF uptake and intracellular distribution. Under this dosing regimen, 4E6 was also internalized and co-localized with the previously added PHF (Fig. 11e–h). However, a different pattern was observed in the PHF + Ab group. Under these conditions, PHF and 4E6 were also seen co-localized, but the PHF-antibody complexes were extracellular (Fig. 11i–l). These results indicate that although these two dosing methods are effective in reducing pathological changes associated with PHF addition, the mechanism of action differs. When cells were incubated with 4E6 prior to PHF, PHF puncta could be seen in the cells, but 4E6 was not apparent, indicating that the antibody may have been cleared from the cell (Fig. 11m–p). These results help to explain the inefficacy of this paradigm.

We also examined binding between 6B2 and PHF. In the PHF → Ab condition, intracellular colocalization was observed, however, in the PHF + Ab condition the large extracellular PHF-antibody complexes were not seen (Fig. 11q–x). Rather, we observed intracellular PHF material, but 6B2 was not present. In the 6B2 → PHF cells, again the PHF material was clearly visible, but the antibody was not (Fig. 11Y-BB).

#### 4E6 recognizes primarily solubilized PHF whereas 6B2 binds mainly to aggregated PHF and sarkosyl insoluble tau protein

To further clarify these intriguing results, binding of the antibodies to tau peptides, solubilized PHF and other tau fractions, was characterized in BIACORE, dot blot and ELISA assays.

#### BIACORE assay

Binding to the P-Ser396 peptide was not detected for either antibody. However, for all other epitope peptides, 6B2 yielded K_D_ values substantially lower (10^−9^-10^−10^ M) than those seen with 4E6 (10^−7^ M), indicating much higher affinity for the immunogen epitope (Table 1).

**Table 1:** Antibody binding to tau peptides

K_D_ (BIACORE)	4E6	6B2
30 amino acid peptides		
Tau379-408 [P-Ser396/404]	2.71 × 10^−7^	3.95 × 10^−10^
Tau379-408	2.12 × 10^−7^	2.51 × 10^−9^
23 amino acid peptides		
Tau386-408 [P-Ser396/404]	4.69 × 10^−7^	2.39 × 10^−9^
Tau386-408 [P-Ser404]	2.78 × 10^−7^	4.11 × 10^−9^
Tau386-408 [P-Ser396]	ND	ND

The binding of 4E6 and 6B2 to tau peptides corresponding to the 396/404 region of the tau protein was examined using a BIACORE assay. Neither antibody showed binding to the P-Ser396 which differs from previously published ELISA assays [11]. Using ELISA, 4E6 bound very poorly to the P-Ser396 peptide coated onto the plate, however, 6B2 did show binding. This may be due to conformational changes which occur in the peptide when binding to the plate, or differences that occur when the antibody is immobilized. We observed similar lack of binding of 4E6 and 6B2 to the P-Ser396 peptide in solution in competition ELISAs (data not shown), confirming the accuracy of the BIACORE data. These findings emphasize that a variety of methods should be used when assessing antibody affinity

#### Dot blot assay

**Fig. 12:**
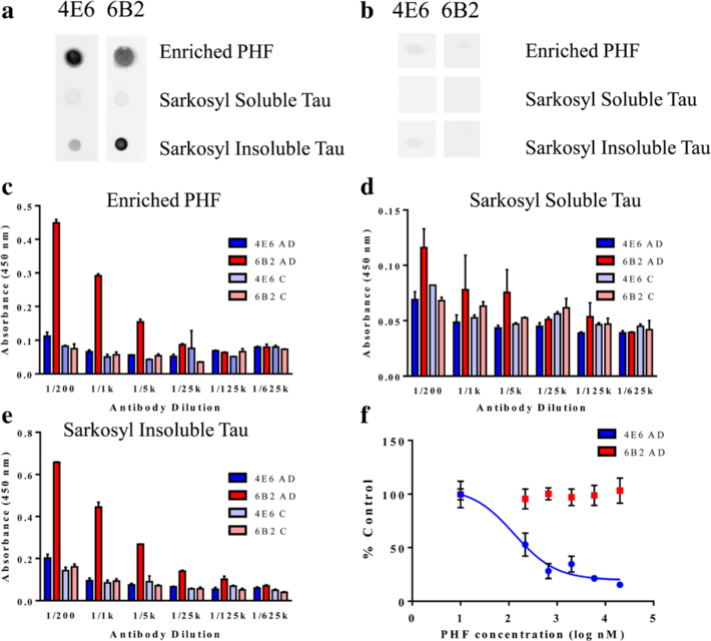
4E6 and 6B2 differ in their binding to human derived PHF material. **a** Different tau species were spotted onto nitrocellulose and incubated with either 4E6 or 6B2 as the primary antibody. 4E6 bound better to solubilized PHF but 6B2 reacted more strongly with the sarkosyl insoluble tau fraction. Both antibodies had limited reactivity with sarkosyl soluble tau protein. **b** The same three tau fractions were prepared from control brain, and spotted onto nitrocellulose. Neither antibody showed binding to the sarkosyl soluble fraction, and only limited binding to the solubilized PHF and sarkosyl insoluble tau. (Images for all three samples for the 4E6 and 6B2 treated control brains were taken from the same strip, the order has been changed for clarity.) **c** Plates were coated with solubilized PHF from AD and control brains. 6B2 showed significantly higher binding to AD than control, and than 4E6 to either AD or control at dilutions, 1/200-1/125 (*p* < 0.0001–0.05). 4E6 did not show significantly higher binding to AD versus control. **d** The assay plate was coated with sarkosyl soluble tau from AD and control brains, and serial dilutions of 4E6 and 6B2 were added. At the 1/200 dilution 6B2 showed significantly higher binding to AD than control, and higher binding than 4E6 to either fraction (*p* < 0.01, 0.05 and 0.05 respectively). **e** Assay plates were coated with sarkosyl insoluble tau. 6B2 showed significantly higher binding to AD relative to control, and than 4E6 to either AD or control, from dilutions 1/200-1/125 k (*p* < 0.0001–0.05). As before, no significant differences between AD and control sample were seen with 4E6. **f** Competitive ELISA assays were performed by pre-incubating the antibodies with increasing concentrations of solubilized PHF material (0.01 -1 μg/ml). 6B2 binding was not inhibited at any PHF concentration. However, 4E6 binding was inhibited in a dose-dependent manner with an IC50 of 71 nM. All of the results show that 4E6 preferentially binds solubilized tau species, while 6B2 primarily binds to insoluble highly aggregated tau. All columns or points on each graph have SEM error bars, however some of those are too small to be visible

Binding of 4E6 and 6B2 to PHF was first assayed using a dot blot of different tau fractions (Fig. 4, Fig. 12). The solubilized PHF-, sarkosyl soluble- and sarkosyl insoluble fractions from the same human AD brain were applied to nitrocellulose membrane, which was then incubated with 4E6 or 6B2 (Fig. 12a). 4E6 had higher affinity for solubilized PHF but 6B2 bound better to the sarkosyl insoluble fraction. Neither antibody bound well to the sarkosyl soluble fraction. Both 4E6 and 6B2 showed limited binding to control samples using dot blot (Fig. 12b). No visible reactivity was seen in the sarkosyl soluble fraction, and only minimal reactivity in the other two fractions. Note that the control tissue had very limited if any pathological tau and the pelletable material was much less than in the AD tissue and likely contains various proteins. Same amount of protein was blotted for AD and control tissue.

#### ELISA assays

Two different ELISA assays were performed to assess binding of 4E6 and 6B2 to different tau fractions from AD and control brain. In the first, the plate was coated with material from either the sarkosyl soluble, solubilized PHF or sarkosyl insoluble fractions (1 μg/well) and dilutions of antibody were added.

When plates were coated with the solubilized PHF (Fig. 12c), 6B2 showed significantly higher binding to wells coated with material from AD brain than control for all dilutions up to 1/125 k (*p* < 0.0001–0.05) and significantly higher binding that 4E6 to either AD or control at 1/200 - 1/5 k (*p* < 0.0001). In contrast, 4E6 did not show significantly higher binding to AD tau versus control tau at any of the dilutions.

In plates coated with sarkosyl soluble tau (Fig. 12d), low binding was detected even at the highest antibody concentrations. At the 1/200 dilution, 6B2 showed significantly higher binding to AD tau than tau from control brain, and also higher binding than 4E6 to either AD or control tau (*p* < 0.01, 0.05 and 0.05 respectively). None of the other dilutions or conditions showed any significant differences between samples.

Finally, when the plates were coated with the sarkosyl insoluble tau (Fig. 12e), 6B2 showed significantly higher binding than 4E6 to AD tau at the 1/200-1/125 k dilutions (*p* < 0.0001–0.05) and also significantly higher binding to AD tau at the same dilutions (*p* < 0.0001–0.05). Again, there was no significant difference in binding to AD versus control with 4E6.

A competition ELISA was then performed to determine antibody binding to PHF in solution. In this assay, plates were coated with solubilized PHF as described above, but before antibodies were added aliquots were incubated for 1 h with increasing concentrations of solubilized PHF (0.01–1 μg/ml). Under these conditions, binding to solubilized PHF markedly inhibited binding of 4E6 to the PHF coated onto the plate, but 6B2 binding was not affected. At the highest PHF concentration, 4E6 binding to the wells was reduced by 85 %. The IC50 value was determined to be 71 nM (Fig. 12f). In contrast, 6B2 did not show reduced binding to the wells at any PHF concentration. These data indicate that the two antibodies are binding to different tau species within the AD-derived PHF material; 4E6 to solubilized PHF and 6B2 to aggregated PHF.

Alone, the ELISA and dot blot binding data are of limited utility for clarifying the efficacy or lack thereof of the antibodies. However, combined with findings from the confocal (Fig. 11l vs. x) and biochemical analyses (Fig. 3g) suggests that efficacy of 4E6 and lack thereof for 6B2 may be explained by the degree of interaction/neutralization of PHF. Dot blot assay and ELISA data show that although both 4E6 and 6B2 bind to PHF, the affinity and preferred species differs, with 6B2 potentially binding to a more highly aggregated but less toxic tau form (Fig. 12a). In contrast, in all assays 4E6 showed less binding to aggregated tau and preferentially binds to the solubilized PHF. Despite strong binding to the solubilized PHF fraction on dot blots relative to control samples, in ELISA assays 4E6 shows a limited ability to bind to the tau which is aggregated onto the wells. Ab-PHF complexes are not seen with the co-incubation of 6B2 and PHF because the latter is in its solubilized form in the culture media which does not bind 6B2 (Fig. 12f). (The solubility of the PHF fraction under experimental concentrations was confirmed using ultra-centrifugation, as described in the Methods section.) pH changes in endosomes/lysosomes may promote aggregation, resulting in a mixture of soluble and insoluble forms of PHF and, therefore, binding of both antibodies in these vesicles (the PHF → Ab condition). However, only binding of 4E6 to the solubilized PHF form is beneficial whereas 6B2 binding has no effect. This detailed clarification has major implications for the development of tau immunotherapies and for understanding the pathogenesis of tauopathies.

#### 4E6 reduces the spread of pathological tau between cell populations

**Fig. 13:**
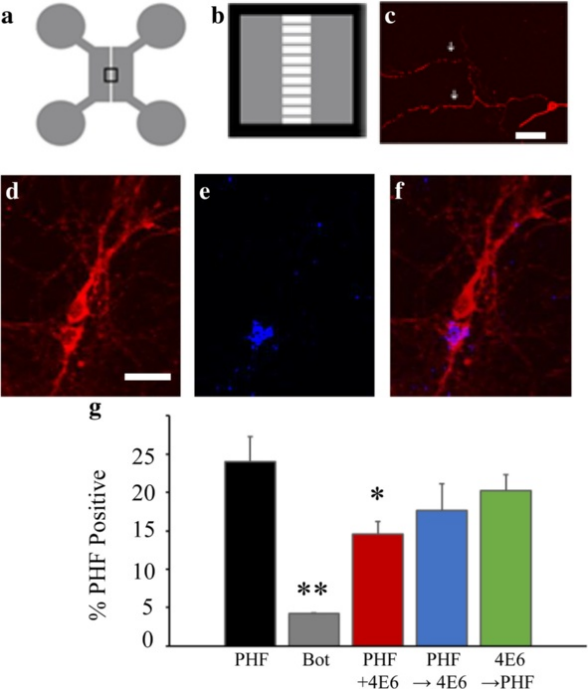
4E6 reduced the spread of tau between neurons. **a**, **b** Schematic of microfluidic chambers, showing the reservoirs that the cells were added to. *Panel B* is a magnification of the box in *panel A* showing the microgrooves which connect the two reservoirs. **c** Confocal image showing axons growing through the microgrooves. Cell is stained with pan-tau antibody. (scale bar = 150 μm) **d**–**f** Fluorescently labeled PHF material (1 μg/ml) was added to the chamber containing JNPL3 cells. Coverslips were fixed and stained with an antibody recognizing total tau. Stained wild-type neurons from the opposite chamber are visualized in **d**, and one of them has prominent PHF puncta in the cell body as seen in E. Merged image of **d** and **e** is depicted in F (scale bar = 50). **g** Neurons in the first chamber were treated with PHF and 4E6 in combination as described in Fig. 4b. Following addition of the antibody, cells were incubated for a further 72 h. Number of cells in the opposite side containing PHF puncta was recorded. Botulinum toxin was used as a negative control. In the PHF alone condition, 24 % of cells were PHF positive. Incubation with botulinum toxin reduced this percentage to 4 % (*p* < 0.01). In the PHF + Ab group this was reduced to 14.6 % (*p* < 0.05), and 17.6 % in the PHF → Ab condition. *: *p* < 0.05, **: *p* < 0.01

The ability of 4E6 to prevent the spreading of PHF between cells was assessed using microfluidic axon isolation chambers (Fig. 13a–c shows a schematic of the chambers and neuron growing in the device). Briefly, Tg cells in one chamber were treated using one of the dosing paradigms described above and the percentage of PHF positive WT cells in the opposite chamber was determined (Fig. 13d–f show PHF positive WT cells).

A one-way ANOVA showed a significant treatment effect (*p* = 0.002; Fig. 13g). In the PHF alone treated cultures, 24 ± 3 % of the WT cells contained fluorescently labeled PHF material. When 50 nM of botulinum toxin was added to PHF treated cultures, this was reduced to 4 ± 0.2 % (*p* < 0.01), indicating that the PHF in the WT cells gets there via synaptic release. PHF + Ab also significantly reduced the percentage of PHF positive WT cells to 15 ± 2 % (*p* < 0.05). PHF → Ab treatment groups showed a lower percentage of PHF positive WT cells, 17 ± 4 % although the results did not reach significance. However, as expected based on other results, there was no significant change in the percentage of PHF positive WT cells under Ab → PHF conditions, further confirming the inefficacy of this approach.

#### 4E6 is effective intra- and extracellularly in blocking PHF-induced toxicity and associated tau pathologies

**Fig. 14:**
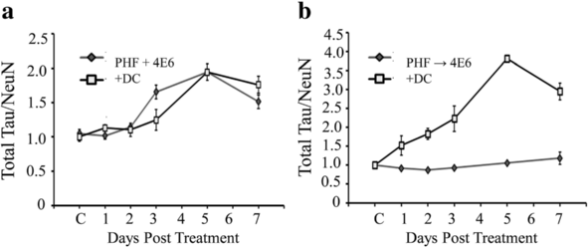
Mechanism of action is influenced by dosing method. PHF (10 μg/ml) was added to JNPL3 neurons, with 4E6 added either at the same time or 24 h later. In additional cultures, 1 μg/ml dansyl cadaverine (DC), an inhibitor of clathrin mediated endocytosis, was also added along with 4E6 to determine whether antibody internalization is necessary for 4E6 to exert its effects. **a** In the PHF + Ab treatment group, addition of DC did not affect the NeuN corrected tau levels, indicating that the antibody is working extracellularly. **b** When DC was present in the PHF → Ab condition, total tau/NeuN ratios was shifted beginning at 24 h, and the relative tau levels were significantly higher after 7 days. Hence, under these conditions, the antibody is working intracellularly

To further investigate whether timing of antibody and PHF affect the mechanism of action, an additional group of cells was plated and dosed as described above. However, when 4E6 was applied to the cultures, 1 μg/ml of dansyl cadaverine (DC) was added as well (Fig. 14). DC is an inhibitor of clathrin (receptor)-mediated endocytosis and in previous experiments, not using exogenous PHF, has been shown to block uptake of 4E6 [10]. Thus, these experiments allowed us to confirm whether antibody internalization is required to prevent PHF-induced pathology. When total tau levels were examined by immunoblot, there was no significant difference between samples incubated with or without DC under either dosing conditions (data not shown). In PHF + Ab samples, addition of DC did not change NeuN levels (data not shown) or tau/NeuN ratio (Fig. 14a). However, when cells in the PHF → Ab condition were incubated with DC, a significant decrease in NeuN was observed when compared to cells without DC (NeuN reduced to 32 % of control, *p* = 0.00005). Further, the ratio of Tau/NeuN was significantly shifted in the PHF → Ab samples where DC was added (1.18 for cells without DC and 2.95 for DC treated cells, *p* = 0.008; Fig. 14b). These results indicate that under conditions where 4E6 and PHF are added together, receptor-mediated internalization (via Fc receptors based on our prior findings [10]) is unnecessary for the antibody to prevent PHF induced pathological changes. In contrast, once the PHF material has been taken up by the neurons, blocking antibody internalization reduces its efficacy in preventing PHF toxicity.

## Discussion

Our findings indicate that two antibodies against the same epitope region have very different effects on cognition in a mouse model of early stage tauopathy. Both antibodies have phospho-selectivity for the immunogen but differ in many ways. Interestingly, the lower affinity antibody, 4E6, is effective in acutely improving spatial learning and memory and reducing soluble phospho-tau, whereas the higher affinity antibody, 6B2, is ineffective. Importantly, we further show identical efficacy differences in a primary neuronal tauopathy culture model treated with paired helical filaments (PHF) isolated from an Alzheimer brain. This indicates that the ex vivo culture model has similar predictive validity as the mouse model although the measured parameters are not comparable.

Modest tau pathology was detected by immunohistochemistry in brains of htau mice, and the treatment and control groups did not appear to differ. Under such conditions of early stage tau pathology, it is easier to quantitate early tau pathology on western blots than by immunohistochemistry, and on such blots insoluble tau protein was clearly present in the 12–13 month old htau mice. Neither tau antibody induced changes in insoluble tau levels as measured by human specific tau antibody (CP27), although 4E6 markedly improved spatial learning and memory. Analyses of the soluble tau fraction revealed that these cognitive benefits were associated with reduced levels of phospho-tau protein (PHF-1 reactive). It is likely that under such acute treatment conditions, global changes in insoluble tau levels may not be readily achievable, whereas soluble pathological tau protein should be more amenable to clearance. Indeed, the PHF-1 antibody recognizes a phospho-tau epitope within the same region as 4E6, which may explain why this tau fraction is preferentially cleared. However, it does not appear to be oligomer specific clearance, as we did not observe any differences in T22 immunoblots between 4E6 and IgG-treated mice.

We did not observe a functional rescue of associative fear memory following acute treatment with either 4E6 or 6B2. There are many possible explanations for this. First, the training protocol used may have been too ‘strong’ to detect a subtle memory deficit. This may be particularly important because the overall tau pathology that we observed, although present, was mild. In our prior studies we only detected associative memory deficits in aged mice with greater levels of tau pathology [24].

Despite the different model systems used, the findings obtained from ex vivo and in vivo experiments are consistent and not model dependent, which supports their validity. In both cases, 4E6 shows efficacy in preventing tau pathology and associated toxicity/cognitive impairment, while 6B2 does not.

An insight into the relevant tau species was obtained from ELISA and dot blot studies of antibody binding to soluble, solubilized, or aggregated human tau species. 4E6 recognizes primarily solubilized PHF, in an ELISA and dot blot assay, which may explain lack of more global tau changes in the animals under such acute conditions. Mice at this age with modest tau pathology may be ideal to assess acute effects of therapies, particularly under pairwise cognitive comparison as used herein, which improves the sensitivity of detecting beneficial effects. Such in vivo learning and memory benefits by 4E6 and lack thereof for 6B2 in the htau mice are in agreement with the efficacy results in vivo and in the tauopathy culture model. Interestingly, soluble tau species have recently been linked to LTP and memory in htau mice [25].

Although the ELISA and dot blot assays provided useful information on the tau binding properties of 4E6 and 6B2, the data obtained from confocal imaging was of greater value in determining mechanism and possible explanations for the differences in efficacy. With co-incubation in the culture assay, extracellular complexes of exogenous PHF and 4E6 formed (Fig. 11l) as 4E6 binds to soluble PHF. This complex formation neutralized PHF and prevented its uptake. However, with 6B2, such complexes did not form, as 6B2 does not bind well to solubilized PHF, and PHF was detected intraneuronally (Fig. 11x). This indicates that 6B2 could not prevent PHF uptake and toxicity. These results support that antibodies can be beneficial while working in the interstitial space between cells. In the living brain, these tau-antibody complexes could then be taken up and cleared by microglia as we have seen previously [11] and others have studied more extensively [26, 27].

Alone, addition of 10 or 1 μg/ml PHF dose-dependently induced cell loss, as measured using LDH and NeuN levels, as well as increased total and phosphorylated tau in the remaining neurons. It spread between cell populations, through release and subsequent uptake by other neurons. To test the efficacy of our antibodies, we utilized three different dosing methods differing in the timing of tau and antibody administration. For one of the antibodies, 4E6, two of these methods, addition of the PHF and antibody together, and addition of 4E6 24 h after PHF, prevented PHF toxicity, seeding, and spread. Interestingly, although similarly effective, the mechanism through which the protection occurs differed between the dosing paradigms.

When 4E6 or 6B2 were added 24 h after PHF, these colocalized intracellularly with PHF but only 4E6 prevented PHF toxicity. Based on the confocal data from the co-incubation experiments, as well as dot blot and ELISA data, 4E6 binds better to solubilized PHF than 6B2 which reacts better with aggregated PHF and insoluble tau. Hence, this feature may explain the intracellular efficacy of 4E6. It may prevent PHF polymerization, facilitating access of lysosomal enzymes to clear PHF and/or directly neutralizing soluble PHF and preventing toxicity. However, 6B2-PHF binding may be inert without promoting disassembly. Furthermore, due to poor binding it may be unable to prevent PHF fibril formation and/or toxicity of soluble PHF.

Together, these findings explain the therapeutic efficacy of 4E6. It is capable of both extracellular blockage and intracellular clearance of PHF. Our previous data indicates that 4E6 enters the endosomal/lysosomal system within tauopathy neurons and promotes clearance of native tau, possibly by preventing aggregation [10, 11, 13]. Other groups have observed internalization of tau antibodies [9, 12], and lysosomal colocalization [9]. Further, neuronal colocalization between antibody, target, and endosomal/lysosomal markers has been seen for α-synuclein antibodies in a PD mouse model [28]. In other experiments, tau antibodies are able to block the uptake of pathological tau or improve experimental outcomes without apparently entering neurons [15–17]. Whether antibodies are taken into neurons is likely influenced by several factors including, charge, target and Fc receptor affinity, and as data presented herein suggests, location of the target and timing of antibody addition.

In contrast, pre-incubation with 4E6 was ineffective at reducing toxicity or seeding of tau pathology. A likely reason is the relative lack of the target epitope under these conditions. Previously, we showed that neuronal 4E6 uptake correlates highly with pathological intracellular tau levels [10]. When the antibody is added first, efficacy requires retention in neurons until PHF addition 24 h later. However, a shortage of the target means the antibody will remain unbound, and more prone to degradation or recycling out of the cell, as seen via confocal imaging. Although 4E6 was ineffective under these conditions, it does not rule out prophylactic administration of tau antibodies, as circulating antibodies could prevent disease initiation by clearing early-stage tau aggregates. Exogenous antibodies have a half-life of one to three weeks and lower doses could be used in pre-symptomatic individuals at risk.

Notably, the different efficacies between dosing methods were also observed in the spreading assay using the microfluidic chambers. In both model systems, only the co-incubation dosing or PHF → Ab was effective, whereas Ab → PHF was not.

In our previously published findings [11], both 4E6 and 6B2 showed efficacy in preventing increased phospho-tau levels in a brain slice model, in contrast to 6B2’s ineffectiveness in the primary neurons in the current study. There are likely multiple factors which contribute to these differences. In the slice culture system, treatment with antibodies lasted for up to 6 weeks and no exogenous tau was introduced in that system. In the present experiments, a much shorter time scale is used (7 days as opposed to six weeks) and we are utilizing PHF tau isolated from a human AD brain. The tau found in the PHF fraction also represents a different stage of tangle formation. Furthermore, the PHF isolated from the AD brain may have additional posttranslational modifications not present in the endogenous tau of the slices. Differences in cell health over the course of the experiments are likely also influenced by the culture model. In the primary cultures, neurons lack the trophic support provided by glial cells, which are present in the brain slices.

We have shown that antibody uptake into neurons can be blocked with an antibody against FcII/III receptors or with dansyl cadaverine, which blocks receptor-mediated endocytosis [10]. Under the co-incubation conditions, blocking antibody uptake had no effect on the outcome. However, when 4E6 was added 24 h after PHF addition, blocking its uptake prevented its beneficial effects. These findings confirm that under co-incubation conditions, the antibody is working extracellularly but when it is added 24 h after PHF, its effects are intracellular.

We have previously shown that both antibodies are taken up into tauopathy neurons in brain slice- and primary cultures, in which they colocalize with tau aggregates in the endosomal-lysosomal system [10, 11]. Furthermore, 6B2 and its single chain variable fragment derivative can be used to image tau lesions in vivo and end up in the same neuronal compartments after peripheral injection [14]. Such uptake and colocalization is by itself not an indication of efficacy but we have shown that prevention of 4E6 neuronal uptake blocks acute antibody-mediated tau clearance [10]. The culture data confirms such intracellular clearance and additionally shows prevention of neurotoxicity by 4E6 in a different culture model, which is more relevant to human disease as AD derived PHF material is used. Furthermore, in our PHF-treated primary culture study, 6B2 was ineffective under various experimental conditions using multiple outcome measures. Overall, 4E6 may be better suited as a therapeutic antibody targeting soluble tau species and 6B2, or ideally its smaller derivatives with better access to the target, useful as an imaging probe for insoluble tau lesions.

Specifically, the ex vivo culture model shows that 4E6, a monoclonal tau antibody targeting the phospho-serine 396/404 region prevented toxicity and reduced tau levels induced by the addition of Alzheimer’s brain-derived PHF material. Importantly, another tau monoclonal, 6B2, which has substantially higher affinity for the tau peptide immunogen and aggregated PHF tau than 4E6, was ineffective under these experimental conditions. Further analyses revealed that 4E6 had higher affinity than 6B2 for the solubilized PHF that was used to promote toxicity in cultured neurons. This likely explains the efficacy of the former antibody and lack thereof for the latter. These findings have major implications for the development of passive tau immunotherapies. Efficacy cannot be predicted by affinity to the immunogen alone or to aggregated tau, but has to be determined in biological models of tau pathology. Combined with imaging data, these results provide information on how affinity and efficacy relate.

Other tau immunotherapy studies have reported efficacy differences between antibodies recognizing epitopes of different sequences of tau and one study between different isotypes of two antibodies of similar affinity against the same epitope (for review see [5]). 4E6 and 6B2 are of the same isotype, IgG1, and our findings show for the first time that subtle difference in epitope recognition can profoundly affect efficacy. Importantly, we have confirmed and provided mechanistic insight into these in vivo differences in a disease relevant ex vivo neuronal culture model, in which we promote tau pathology with Alzheimer’s brain-derived PHF in primary neurons expressing familial tau mutation. Hence, the contrasting efficacies are seen consistently in different models with or without tau mutation and may have major therapeutic implications for both familial and sporadic tauopathies. The models employed have strong construct and face validity as they are based on sound theoretical rational as normal or familial (mutated) human tau is being expressed, and have the key features associated with tauopathies, namely tau aggregation, toxicity, and associated cognitive impairments in the animals. The human PHF culture model has strong predictive validity for the outcome in the animal model, but it remains to be seen if this holds up in clinical trials.

## Conclusions

Overall, these findings indicate that antibody efficacy cannot be predicted by affinity to the immunogen alone or broad reactivity with various typical pathological forms of tau but has to be determined in biological models of tau pathology. Binding to solubilized PHF rather than aggregated PHF or insoluble tau predicts efficacy in these ex vivo PHF seeding and spreading culture models and in a tauopathy mouse model. 4E6 promotes clearance of pathological tau and thereby prevents its toxicity via intra- and extracellular pathways. Such wide spectrum antibodies are likely to be more efficacious than antibodies that can only work outside neurons. Importantly, the ex vivo findings fit nicely with acute antibody efficacy in vivo in improving cognition, which was associated with clearance of soluble phospho-tau.

Future studies should consider that relatively subtle epitope differences, even within the same tau region, can have major implications for therapeutic outcome, and that higher affinity for the immunogen or various pathological forms of tau does not necessarily translate into better efficacy. Considering that antibody humanization and scale up for clinical trials may alter binding characteristics compared to the original mouse monoclonal antibody, these findings may have major implications for ongoing and future clinical trials of tau antibodies.

## Methods

### Animals and in vivo experimental design

#### Ex vivo

Pups from the homozygous JNPL3 mouse line (human 0N4R with P301L mutation, Taconic) were collected at postnatal day zero for primary cultures [29]. This model was chosen because of its robust tau expression and phosphorylation even at an early age.

For tau spread experiments, wild type (WT) pups from the same strain background were also utilized. All breeding animals were monitored closely during the progression of pregnancy to ensure that pups were collected on the day of birth to make certain that each culture was at the same developmental age when plated.

#### In vivo

The htau model (Jackson Laboratories, stock#004808; [20]), expresses all six isoforms of unmutated human-tau protein on a null mouse-tau background and develops progressive tau pathology. This model was utilized in the whole animal treatment studies due to its more naturalistic tau expression relative to other tauopathy models. It was used to assess therapeutic benefits of acute passive immunotherapy with tau monoclonal antibodies 4E6G7 (4E6) or 6B2G12 (6B2), purified from our hybridoma by Genscript (Paramus, NJ), relative to IgG-injected (Equitech Bio Inc.) controls. In the 4E6 study, 18 mice (7 males (M) and 11 females (F)) received 4E6 and 18 mice (8 M and 10 F) control IgG. No animals died during the study but, four mice in each group were excluded from all analysis, due to no human-tau expression, despite containing the transgene, leaving 14 4E6 mice (4 M and 10 F) and 14 IgG mice (7 M and 7 F) for analysis.

In the 6B2 study, only female mice were enrolled, 12 per group. As in the 4E6 study, no animals died during the experiment, but four IgG-treated and one 6B2-treated mouse were excluded due to lack of human-tau expression, leaving 11 6B2 treated mice and 8 IgG controls for analysis.

At the start of the study, the htau mice were 11–12 months of age and split into two groups with similar cognitive and motor status before receiving three antibody injections and going through retesting on the same behavioral tests and an additional fear-conditioning test, followed by brain extraction for tissue analysis. The mice went through adaptation and pre-tests using Rotarod, Open Field and Closed Field Symmetrical Tests on days 1–10 as we have detailed previously [30], followed by antibody injection on days 11 and 14 and retesting on days 15–18. Third injection was delivered on day 24 followed by fear-conditioning test on days 27–28, and perfusion on day 30.

All animals were housed at NYU School of Medicine animal facilities and cared for by the veterinary staff in AAALAC-approved facilities. All the procedures were approved by the Institutional Animal Care and Use Committee (IACUC) committee of the university, and are in accordance with NIH Guidelines, which meet or exceed the ARRIVE guidelines.

### Isolation of paired helical filaments (PHF)

PHF tau was extracted from the brain of a human AD patient as described by others [23] but with a few modifications (See Fig. 4) to create the three fractions used to treat cells and assess antibody binding via dot blot. Briefly, the tissue was homogenized in buffer (pH 6.5; 0.75 M NaCl, 1 mM EGTA, 0.5 mM MgSO_4_, and 100 mM 2-(*N*-morpholino) ethanesulfonic acid) and centrifuged at 11,000 x g for 20 min to remove debris. The supernatant was centrifuged for an additional 60 min at 100,000 x g. The resulting pellet was resuspended in extraction buffer (10 mM Tris; 10 % sucrose; 0.85 M NaCl; and 1 mM EGTA, pH 7.4) and centrifuged at 15,000 x g for 20 min. The supernatant was retained and incubated with 1 % sarkosyl at room temperature. This supernatant was then centrifuged for 60 min at 100,000 x g. The supernatant from this step was retained and designated the sarkosyl soluble fraction. The pellet was resuspended in 50 mM Tris–HCl buffer and designated the sarkosyl insoluble fraction. For the cell culture experiments this fraction was first heated briefly to 37 °C and then dialyzed in PBS, yielding the enriched PHF fraction. The heating and dialysis promote solubility and result in solubilized enriched PHF tau fraction which was used in the experiments. Under our conditions, the PHF fraction is soluble at the doses used (1 and 10 μg/ml). Prior work by others indicates that PHF can be soluble at least up to 100 μg/ml [23]. In addition, other groups have demonstrated that dilution of tau filaments leads to their disassembly [31–33].

In order to assess the solubility of the PHF material at the concentrations used, we prepared a 10 μg/ml solution and subjected it to centrifugation at 100,000 x g for 60 min. Following this spin, the supernatant was removed. Under these conditions, no visible pellet was seen.

Brain tissue from a control brain was processed in the same way. Note that the control tissue has very limited if any pathological tau and the pelletable material is much less than in the AD tissue and likely contains various proteins. Same amount of protein was used in each assay for AD and control tissue.

### ELISA assays

ELISA assays were performed as described [11]. Briefly, 96 well plates were coated with one of three different tau fractions (sarkosyl soluble, solubilized PHF, or sarkosyl insoluble) derived from either AD or control brains dissolved in 50 mM carbonate buffer (pH 9.6). Plates were blocked using Superblock (Fisher Scientific) for one hour at room temperature. After blocking, serial dilutions (1/200-1/625,000) of the antibodies were added to the plate and incubated for 2 h at room temperature. All antibodies were adjusted to a concentration of 1 mg/ml before dilution. Plates were washed with 0.1 % TBS-T and HRP-conjugated mouse secondary antibody (1:5000) was added for one hour. TMB peroxidase EIA reagent (Fisher Scientific) was used to develop the signal and stopped with 2 N sulfuric acid. Absorbance at 450 nm was read using a BioTek Synergy 2 plate reader.

For the competitive ELISA assays, a single antibody concentration was utilized (1:1000 dilution of 1 μg/ml stock). Aliquots of 4E6, 6B2 and IgG1k isotype control (eBioscience) were incubated for 30 min with increasing concentrations of the human derived solubilized PHF material prior to plating.

### Fluorescent labeling

4E6 and human derived solubilized PHF material were labeled using Alexa Fluor 488 and 647 labeling kits, respectively as per kit instructions.

### Primary neuronal cultures

Cultures were prepared as described from the cortex and hippocampus of p0 pups, with all components purchased from Invitrogen unless otherwise detailed [10]. After 24 h in culture, plating media was removed and neuronal media (Neurobasal A, 1 ml B27, 17 μl basal medium Eagle) added. As in previous studies, the purity of the neuronal cultures was assessed using confocal imaging. Under these conditions, about 95 % of cells are neurons (positive for NeuN) [34].

For cultures grown in microfluidic chambers the same procedure was utilized. Cells from the JNPL3 mice were plated on one side of the axon isolation device, and were allowed to incubate for 72 h. Following this period, brains from WT animals were harvested and those cells plated on the opposite side.

### Ex vivo experimental design

Primary neuronal cultures were prepared as described and allowed to recover in culture for one week prior to treatment [10]. Cells were incubated with either 1 or 10 μg/ml of the human derived PHF material, with cells and culture media collected at 1, 2, 3, 5 and 7 days. For cultures being treated with a combination of PHF and antibody (4E6, 6B2, or IgG), three different treatment strategies were used. In the first, PHF material was added and allowed to incubate with the cultures for 24 h. Following this period, the cells were washed with neuronal media, and fresh media containing 1 μg/ml of antibody was added (PHF → Ab). In the second, PHF material and antibody were added to the culture media simultaneously (PHF + Ab). The third dosing strategy is the inverse of the first, antibody was added 24 h prior to PHF (Ab → PHF). See Fig. 4 for a summary of dosing methods. In experiments using dansyl cadaverine (DC; Fisher Scientific), the same methods were employed with 1 μg/ml DC added along with the antibody.

### LDH assays

Media was collected from all treatment groups after seven days in culture. LDH levels in the media were determined using a Roche cytotoxicity detection kit. Values obtained from treated samples were compared to media collected on day zero to assess toxicity. Media from a set of untreated cells was also collected to examine the normal changes in cell health over the culture period. Treated samples, controls and blanks were added to a 96 well plate and the detection reagents were added as per the instructions. Plates were incubated for 20 min at 37 °C and read using a BioTek Synergy 2 plate reader.

### Microfluidic chambers and tau spreading

Following the addition of the WT cells, cultures were given one week in culture to stabilize. The same three treatment methods were utilized. PHF and 4E6 (1 μg/ml each) were added to the chamber containing JNPL3 cells, while the opposing chamber containing WT cells was left untreated. As a negative control, one group of cells was incubated with 1 μg/ml of the PHF material and 50 nM of botulinum toxin (generously provided by Dr. Edwin Vasquez-Cintron, NYU School of Medicine). The toxin was chosen due to its ability to prevent membrane fusion and the release of membrane bound vesicles, and thus prevent release of PHF tau into the opposite chamber. In neuronal cultures, botulinum toxin has also been shown to prevent the spread of mutant huntingtin by blocking synaptic vesicle release [35]. In all groups, after the final treatment, cells were maintained in culture for a further 72 h. Coverslips were then fixed and stained for antibodies recognizing total tau. The percentage of cells in the contralateral chamber containing labeled PHF material was determined for each treatment group.

### Immunohistochemistry

#### Animals

Following behavioral testing, mice were anesthetized with ketamine/xylazine (250 mg/50 mg per kg body weight, intraperitoneally (i.p.)), and processed as we have described previously [36]. Immunostaining was performed on the right hemisphere of coronal fixed brain sections (40 μm) with mouse-monoclonal tau antibodies that stain pathological tau, PHF-1(1:1000), against the P-Ser396, 404 epitope and MC1 (1:100) which recognizes a conformational epitope. Both antibodies were generously provided by Peter Davies (Feinstein Institute for Medical Research, Manhasset, NY).

#### Cultures

Performed as previously described [10].

### Immunoblotting

#### Animals

The left hemisphere of the brain was homogenized in (5x vol/w) modified RIPA buffer as described [24]. The brain homogenate was centrifuged (20,000xg) for 20 min at 20 °C and supernatants collected as LSS (Low Speed Supernatant). After protein quantification, the volumes were adjusted for equal protein concentration with dilution in modified O+ buffer as described [24], boiled for 5 min, and loaded onto 12 % polyacrylamide gel. For the sarkosyl insoluble fraction, 10 % sarkosyl solution was added to LSS, and the sample mixed for 30 min at room temperature, then centrifuged at 100,000xg for 1 h at 20 °C. The pellet was then washed in 1 % sarkosyl solution and spun again for 100,000xg for 1 h at 20 °C. It was then air dried for 30 min, mixed with 50 μl of modified O+ buffer, vortexed for 1 min, then boiled for 5 min and denoted the sarkosyl pellet (SP) fraction. The LSS and sarkosyl insoluble fractions were both incubated with CP27. The LSS fraction was also probed for total tau using Tau-5, phospho-tau using PHF-1 and oligomeric tau using T22 [37]. All soluble tau blots were normalized using GAPDH.

#### Cultures

Performed as described previously [10]. Prior to immunoblotting, all samples from control and treated cells were assayed for total protein concentration, and normalized accordingly. Neuronal cultures were probed with antibodies recognizing neuronal marker NeuN, total tau (Dako) and tau phosphorylated at Ser199 (SantaCruz). NeuN was used to measure PHF induced cytotoxicity [38–43] and to control for tau levels [44]. Because the expression of tau protein can vary between animals, even when on a homozygous background, each experiment was preformed using cells prepared from a single animal. Untreated control cells for each experiment were also prepared using the same animal. Thus, each experiment has its own unique set of control samples.

### Behavioral studies

#### Sensorimotor tests

##### Rotarod

This test is used to measure forelimb and hindlimb motor coordination and balance. This procedure was designed to assess motor behavior without a practice confound. It was performed similar to as previously described in detail [30].

##### Locomotor activity

This test was performed similarly to those previously described in detail [30].

### Cognitive tests

#### Closed field symmetrical maze

This apparatus is a rectangular field, 65 cm square with 10 cm high walls divided into 36 squares. Two boxes, (16 × 23 × 10 cm), are attached to the square at its diagonal corners. The symmetrical maze is a modification of the Hebb-Williams and Rabinovitch-Rosvold tests as we have described [30, 45, 46]. Briefly, each end box functions as both a start box and a goal box. The mice run in opposite directions on alternate trials, thereby eliminating intertrial handling which should minimize stress. The barriers are placed in the field in symmetrical patterns, so that mice face the same turns going in either direction within a given problem. On day 0, mice were adapted to a water-restriction schedule (2 h daily access to water) and habituated in the same environment as used for testing. On day 1, all mice were given saccharine-flavored water, for 10 min in each box. On day 2, they were placed in the start box and permitted to explore the field and enter the goal box, in which the saccharine-water reward (0.05 ml) was available. The door to each box was manually opened and closed to allow entry and exit. When the mice were running reliably from the start box to the goal box, they were given four practice trials under the same condition. On day 3, they were given one practice session on a simple problem, in which two barriers were placed in different symmetrical positions in the field so as to obstruct direct access to the goal box. This practice test was repeated for 4 trials. On day 4, formal testing consisted of three barriers graded for the most difficulty (Maze 7), based on our prior findings [30, 45, 46]. Mice were given five trials with an intertrial interval of 2 min. Performance was scored manually by the same observer, who was blinded to the treatment received, in terms of errors (ie, entries and reentries into designated error zones) and time to complete each trial.

In the acute treatment study, mice were then split into control and treatment group, which had similar average tests scores and group variance, taking into account as well their performance on the sensorimotor tests. The mice were retested without practice period after the treatment period.

#### Fear-conditioning

This test was performed as described previously [24]. On the training day, mice were allowed to explore for 2 min in the test chamber. The conditioned stimulus (CS; a white noise 80 dB sound) was presented for 30 s and followed immediately by a mild foot shock (2 s, 0.5 mA) that served as the unconditioned stimulus (US). After 2 min, the mice received a second CS-US pairing. The Freeze Frame monitor system (San Diego Instruments) was used to control the timing of CS and US presentations and to record freezing behavior. During the conditioning procedure, response to the foot shock -typically run, jump, or vocalize- were also recorded. Mice were tested for contextual fear in 3 h for short term memory and 24 h for long term memory, during which mice were placed into the original test chamber for 5 min in the absence of CS and freezing behavior was recorded.

### Statistics

All data were analyzed with GraphPad Prism 6 (San Diego, CA).

#### Ex vivo culture

In the competitive ELISA assay, data from the group that did not receive PHF was used to represent 100 % of the normal binding to the plate, with the other conditions normalized to these values. Values were fitted to a non-linear curve competitive binding formula to determine the IC50 for each antibody. LDH data were analyzed using a one-way AVOVA. Data from PHF and antibody time courses were normalized using untreated cells from the same animal. For each individual time point, the percentage of the control for each sample was determined, and the average and standard error of the mean calculated. For each set of experiments a two-way ANOVA was used to determine the effect of individual treatment group and time. For tau spreading experiments, the percentage of PHF positive cells in each image was determined, and used to determine the average and standard error of the mean for each group. Treatment groups were then compared using a one-way ANOVA. Significant differences between individual time points, and/or groups, were determined using Tukey’s multiple comparisons test.

#### In vivo

The behavioral data with pre- and post-testing (CFSM, Rotorod and Open Field) was analyzed by a paired *t*-test. The Fear Conditioning data and tau levels on western blots were analyzed with an unpaired *t*-test. Two tailed t-tests were used to compare immunoblot results. Welch correction was used if the data failed a test of equal variance. When data failed two out of three normality tests (KS, D′Agostino & Pearson omnibus, and Shapiro–Wilk normality tests) non-parametric Mann–Whitney test was used. The effects of gender and antibody treatment on behavior and immunoblot results were further assessed using a two-way ANOVA.

## Abbreviations

Ab, antibody; ANOVA, analysis of variance; CFSM, Closed field symmetrical maze; DC, dansyl cadaverine; ELISA, enzyme linked immunosorbent assay; IC50: half maximal inhibitory concentration; IgG, immunoglobulin G; K_D,_ dissociation constant; LDH, lactate dehydrogenase; PHF, paired helical filament; Tg, transgenic; WT, wild type

## Additional file

Additional file 1: Figure S1. htau mice show limited tau pathology which is not affected by 4E6 treatment. (PDF 79 kb)
