# Supplementary material for: Affinity of Tau antibodies for solubilized pathological Tau species but not their immunogen or insoluble Tau aggregates predicts in vivo and ex vivo efficacy
Source: Mol Neurodegener. 2016 Aug 30;11(1):62. doi: 10.1186/s13024-016-0126-z (PMC5006503; doi:10.1186/s13024-016-0126-z)
Supplement: Additional file 1: Figure S1. — htau mice show limited tau pathology which is not affected by 4E6 treatment. (PDF 79 kb) [file 13024_2016_126_MOESM1_ESM.pdf]

Supplemental Figure 1. Htau mice show limited tau pathology which is not affected by 4E6 treatment

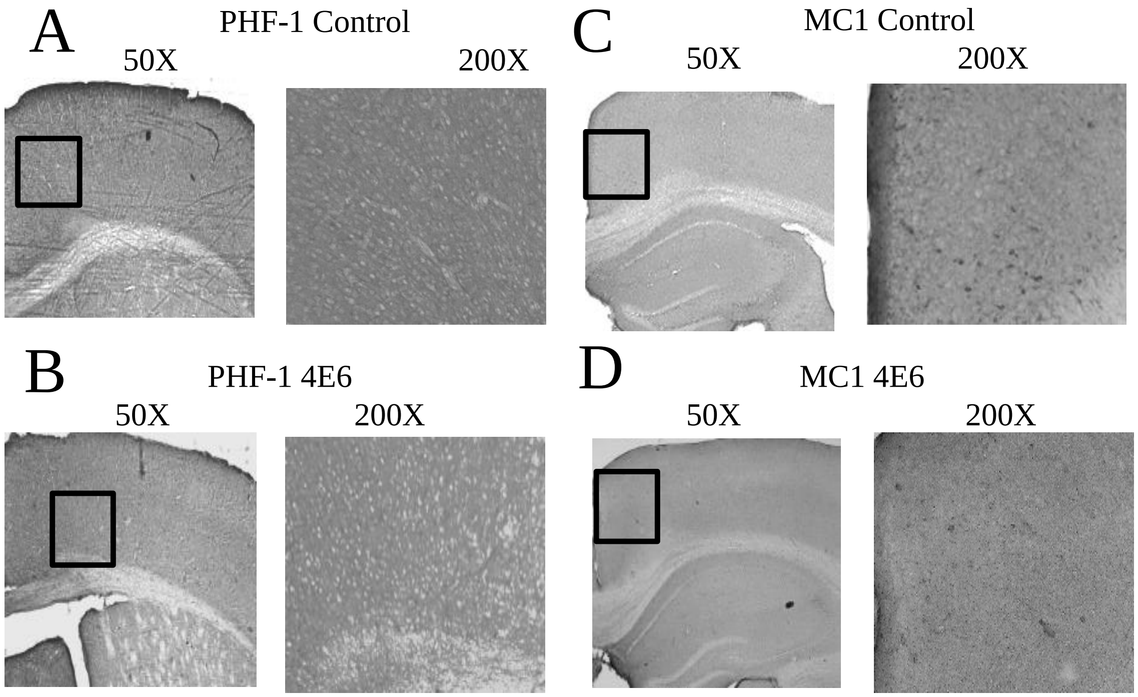

Supplemental Figure 1. Representative images showing tau antibody staining of brain sections prepared from IgG control and 4E6 treated htau mice. **A, B**. Coronal brain section of an IgG control (A) or 4E6 treated (B) htau mouse stained with PHF-1. Some neuropil staining is evident without any cell body staining. **C, D.** Coronal brain section of an IgG control (C) or 4E6 treated (D) htau mouse stained with MC1. Staining is limited as in the PHF-1 stained sections although a few aggregates are evident in the higher magnification images. MC1 is known to detect earlier tau pathology than PHF-1. The box in the left panel (50X magnification of the section, 5X objective) of each image outlines the frame depicted in the right panel (200X magnification of the section, 20X objective)
